# Concurrent Label-Free Mass Spectrometric Analysis of Dystrophin Isoform Dp427 and the Myofibrosis Marker Collagen in Crude Extracts from *mdx-4cv* Skeletal Muscles

**DOI:** 10.3390/proteomes3030298

**Published:** 2015-09-16

**Authors:** Sandra Murphy, Margit Zweyer, Rustam R. Mundegar, Michael Henry, Paula Meleady, Dieter Swandulla, Kay Ohlendieck

**Affiliations:** 1 Department of Biology, Maynooth University, National University of Ireland, Maynooth Co. Kildare, Ireland; E-Mail: sandra.murphy@nuim.ie; 2 Department of Physiology II, University of Bonn, Bonn D-53115, Germany; E-Mails: margit.zweyer@ukb.uni-bonn.de (M.Z.); mundegar@yahoo.de (R.R.M.); dieter.swandulla@ukb.uni-bonn.de (D.S.); 3 National Institute for Cellular Biotechnology, Dublin City University, Dublin 9, Ireland; E-Mails: michael.henry@dcu.ie (M.H.); paula.meleady@dcu.ie (P.M.)

**Keywords:** collagen, Dp427, Duchenne muscular dystrophy, dystrophin, myofibrosis

## Abstract

The full-length dystrophin protein isoform of 427 kDa (Dp427), the absence of which represents the principal abnormality in X-linked muscular dystrophy, is difficult to identify and characterize by routine proteomic screening approaches of crude tissue extracts. This is probably related to its large molecular size, its close association with the sarcolemmal membrane, and its existence within a heterogeneous glycoprotein complex. Here, we used a careful extraction procedure to isolate the total protein repertoire from normal *versus* dystrophic *mdx-4cv* skeletal muscles, in conjunction with label-free mass spectrometry, and successfully identified Dp427 by proteomic means. In contrast to a considerable number of previous comparative studies of the total skeletal muscle proteome, using whole tissue proteomics we show here for the first time that the reduced expression of this membrane cytoskeletal protein is the most significant alteration in dystrophinopathy. This agrees with the pathobiochemical concept that the almost complete absence of dystrophin is the main defect in Duchenne muscular dystrophy and that the *mdx-4cv* mouse model of dystrophinopathy exhibits only very few revertant fibers. Significant increases in collagens and associated fibrotic marker proteins, such as fibronectin, biglycan, asporin, decorin, prolargin, mimecan, and lumican were identified in dystrophin-deficient muscles. The up-regulation of collagen in *mdx-4cv* muscles was confirmed by immunofluorescence microscopy and immunoblotting. Thus, this is the first mass spectrometric study of crude tissue extracts that puts the proteomic identification of dystrophin in its proper pathophysiological context.

## 1. Introduction

Dystrophin proteins exist in a large number of isoforms with greatly differing tissue distributions and are encoded by the largest gene in the human genome within the Xp21 region of the X-chromosome [1,2,3]. The full-length muscle isoform of dystrophin, termed Dp427-M, functions as a membrane cytoskeletal actin-binding protein in contractile fibers [4,5,6]. Dystrophin is tightly linked to the muscle sarcolemma via close interactions with a membrane-associated glycoprotein complex [7,8,9]. Progressive diseases of skeletal and cardiac muscles with primary abnormalities in the dystrophin gene include Duchenne muscular dystrophy, Becker muscular dystrophy, and X-linked dilated cardiomyopathy [10,11,12]. In X-linked muscular dystrophy, the almost complete absence of full-length dystrophin triggers a significant reduction in the dystrophin-associated glycoprotein complex and a plethora of down-stream pathophysiological changes, such as an altered coupling between neuronal excitation and muscle contraction, stretch-induced fiber injury, higher levels of plasmalemmal calcium influx, impaired luminal calcium buffering, and an accelerated proteolytic degradation rate [13,14,15]. In order to elucidate the enormous complexity and potential interconnectivity of these many secondary changes, damage pathways in dystrophic muscles involved in fiber degeneration, inflammation, fatty deposition, and progressive fibrosis are ideally studied by large-scale and comprehensive bioanalytical approaches, such as mass spectrometry-based proteomics [16].

Proteomic profiling is a core discipline of modern systems biology and has been widely applied to basic and applied myology [17,18,19], including the evaluation of the secondary effects of dystrophin deficiency in muscular dystrophy [20]. Both gel electrophoretic techniques and liquid chromatography have been employed for the systematic separation of the assessable skeletal muscle proteome. A variety of changes in skeletal muscle-associated proteins involved in energy metabolism, excitation-contraction coupling, fiber contraction, ion homeostasis, the stress response, and cellular signaling have been identified by mass spectrometry, as reviewed by Dowling *et al.* [21]. This included differential alterations in muscle-associated proteins, such as adenylate kinase isoform AK1 [22], the luminal Ca^2+^-binding protein calsequestrin [23], the cytosolic Ca^2+^-binding proteins regucalcin [24] and parvalbumin [25], carbonic anhydrase isoform CA3 [26], various molecular chaperones and heat shock proteins including αB-crystallin/HSPB5, cvHsp/HSPB7, Hsp70/HSPA and Hsp90/HSPC [27,28,29,30], the cytoskeletal proteins vimentin and desmin [31,32,33], metabolic proteins regulated by PGC1-α [34], the extracellular matrix protein dermatopontin [35,36], and the matricellular protein periostin [37]. Elevated levels of muscle-derived proteins in body fluids have been described for fibronectin, the matrix metalloproteinase MMP-9, creatine kinase, carbonic anhydrase CA3, myosin light chain MLC3, malate dehydrogenase MDH2, transforming growth factor TGFβ1, electron transfer flavoprotein ETFA, fragments of the contractile apparatus-associated protein titin, and the lysosomal-associated membrane protein LAMP1 [38,39,40,41,42,43]. However, the comparative proteomic profiling of crude tissue extracts has not routinely identified the members of the dystrophin-glycoprotein complex, which is probably due to the low concentration and the tight membrane association of this protein assembly [20].

Although dystrophin has been listed in proteomic catalogues describing the overall protein constellation of normal skeletal muscles, the full-length Dp427 isoform of this membrane cytoskeletal protein has not been identified in comparative proteomic analyses using whole tissue preparations [21]. We have therefore attempted the application of sensitive label-free mass spectrometry to evaluate total skeletal muscle tissue extracts from wild type *versus* the dystrophic *mdx-4cv* mouse. The main underlying objective was to analyze in parallel the primary abnormality in muscular dystrophy and the many secondary changes triggered by the deficiency of dystrophin. Previous proteomic studies with a focus on dystrophin have used pre-fractionation approaches, including immuno precipitation, elaborate density gradient centrifugation, and liquid chromatography procedures [44,45,46,47], or specialized mass spectrometric methodology with a stable isotope labelled dystrophin as a spike-in standard for the quantitation of select peptides representing dystrophin within a heterogeneous protein mixture [48]. The recent application of organelle proteomics, in conjunction with label-free mass spectrometry, has succeeded in the identification of dystrophin isoform Dp427, dystroglycan, δ-sarcoglycan, γ-sarcoglycan, and α1-syntrophin by decisively reducing sample complexity using differential centrifugation to enrich the microsomal fraction [49]. However, since subcellular fractionation steps may introduce artifacts in comparative proteomic studies, mainly due to the differences in membrane organization and myofibrosis, the identification of muscle-associated biomarker candidates in crude muscle extracts is more promising for the establishment of a superior marker signature. In addition, the findings from this new study, using whole tissue proteomics, present important verification results in relation to previous subproteomic investigations. Importantly, since skeletal muscles from this animal model of dystrophinopathy are characterized by very few revertant fibers and exhibit myofibrosis [50,51,52,53], the proteomic analysis of total *mdx-4cv* muscle extracts was ideally suited to simultaneously study dystrophin deficiency and secondary fibrotic changes within the same analytical run.

## 2. Experimental Section

### 2.1. Chemicals and Materials

The mass spectrometry-based proteomic profiling of crude tissue extracts from *mdx-4cv versus* wild-type hind limb muscles was conducted using analytical grade reagents and materials obtained from GE Healthcare (Little Chalfont, Buckinghamshire, UK) and Bio-Rad Laboratories (Hemel-Hempstead, Hertfordshire, UK). Ultrapure acrylamide stock solutions were purchased from National Diagnostics (Atlanta, GA, USA). Sequencing grade modified trypsin and Lys-C were from Promega (Madison, WI, USA). Whatman nitrocellulose transfer membranes were obtained from Invitrogen (Carlsbad, CA, USA). The chemiluminescence substrate and protease inhibitors were attained from Roche Diagnostics (Mannheim, Germany). Primary antibodies were purchased from Abcam, Cambridge, UK (ab41803 to annexin 2; ab8592 to desmin; ab11427 to parvalbumin; ab21754 to the β-subunit of tubulin; ab58475 to the α-subunit of the Na^+^/K^+^-ATPase; ab6588 to collagen VI; ab2818 to the fast SERCA1 isoform of the sarcoplasmic reticulum Ca^2+^-ATPase and ab52488 to lactate dehydrogenase). Chemicon International (Temecula, CA, USA) provided peroxidase-conjugated secondary antibodies. For immunofluorescence microscopy, normal goat serum, goat anti-rabbit Alexa Fluor 488, and goat anti-mouse IgG RRX (Rhodamine Red-X) were purchased from Molecular Probes, Life Technologies (Darmstadt, Germany), and Jackson ImmunoResearch (West Grove, PA, USA), respectively. The embedding medium Fluoromount G was from Southern Biotech (Birmingham, AL, USA). A variety of other general chemicals, including bis-benzimide Hoechst-33342, were obtained from Sigma Chemical Company (Dorset, UK).

### 2.2. Animal Model of X-Linked Muscular Dystrophy

Duchenne muscular dystrophy is a monogenic disorder caused by mutations or genetic rearrangements in the 79 exon-spanning gene that encodes the cytoskeletal protein dystrophin [3]. In analogy, the internationally established *mdx* mouse model of dystrophinopathy is almost completely missing the full-length Dp427 isoform of dystrophin due to a point mutation in exon 23 [54,55,56]. Since *mdx* skeletal muscles may exhibit a large number of dystrophin-positive revertant fibers, we have here used the *mdx-4cv* mouse model as an alternative model for studying X-linked muscular dystrophy. Chemical mutagenesis with *N*-ethylnitrosourea has been used to induce a C to T transition at position 7916 in exon 53 [52], generating a nonsense point mutation that leads to premature translation termination [51]. Truncated dystrophin products in mutant mice appear to be highly unstable and are probably quickly degraded in dystrophic muscle tissues, since they cannot be detected by standard immunoblotting [9]. This genetic model has the advantage of displaying 10-fold fewer revertant fibers as compared to the conventional *mdx* mouse [50], which renders it an attractive model for evaluating the efficacy of novel therapeutic strategies [57,58,59]. While the *mdx-4cv* model does not express the full-length Dp427 isoform of dystrophin in either skeletal muscle or brain tissue, it has been shown to express the Dp71 dystrophin isoform in brain tissue at levels comparable to those seen in control mice [53]. This may be due to the fact that the premature stop codon found in exon 53 of the *mdx-4cv* strain is considerably downstream from the internal promoters that give rise to shorter dystrophin isoforms [53]. As a means of investigating altered protein expression in dystrophic muscle, total tissue extracts of hind limb muscles from six-month old *mdx-4cv* mice *versus* age-matched control C57BL6 mice were analyzed, representing a moderate dystrophic phenotype with segmental necrosis [25,31,33]. Fresh tissue samples were acquired from the Bioresource Unit of the University of Bonn [60]. The mice were kept under standard conditions according to German and Irish legislation on the use of animals in experimental research. The animals were sacrificed by cervical dislocation and muscle tissues were isolated immediately. The tissues used for proteomic analysis were quick-frozen in liquid nitrogen and stored at −80 °C prior to analysis.

### 2.3. Preparation of Tissue Extracts from Normal and Dystrophic Hind Limb Muscles

Skeletal muscle samples (100 mg wet weight) from combined muscles of the entire hind limbs (including bulk muscle from the lower and upper leg, but excluding the grasping foot-paw) of six-month-old dystrophic *mdx-4cv* mice (*n* = 4) and age-matched wild-type (*wt*) mice (*n* = 4) were finely chopped and homogenized in 10 volumes of homogenization buffer (20 mM sodium pyrophosphate, 20 mM sodium phosphate, 1 mM MgCl_2_, 0.303 M sucrose, 0.5 mM EDTA, pH 7.0), using a hand-held IKA T10 Basic Homogenizer (IKA Labortechnik, Staufen, Germany) [49]. This buffer was supplemented with a protease inhibitor cocktail from Roche Diagnostics (Mannheim, Germany) to minimize degradation of skeletal muscle proteins [61]. Protein extracts were gently shaken at 8 °C for 2 h using a Thermomixer from Eppendorf (Hamburg, Germany). Following centrifugation at 14,000 *g* for 20 min at 4 °C, the supernatant fractions from *wt* and *mdx-4cv* muscle homogenates were isolated and used for comparative proteomic profiling.

### 2.4. Sample Preparation for Label-Free Liquid Chromatography Mass Spectrometry

Prior to mass spectrometric analysis, total skeletal muscle extracts were purified of interfering agents using the Ready Prep 2D clean-up kit from Bio-Rad Laboratories (Hemel-Hempstead). Subsequent protein pellets were re-suspended in label-free solubilization buffer (6 M urea, 2 M thiourea, 10 mM Tris, pH 8.0 in LC-MS grade water). Following vortexing and sonication, the protein concentration was determined using a Bradford assay protocol [62]. For label-free mass spectrometric analysis, volumes were equalized with label-free solubilization buffer, followed by chemical reduction with 10 mM DTT for 30 min at 37 °C and alkylation with 25 mM iodoacetamide in 50 mM ammonium bicarbonate for 20 min in the dark at room temperature [60]. To quench any remaining unreacted iodoacetamide and limit potential alkylation of trypsin, samples were further reduced with an additional 10 mM DTT for 15 min in the dark at room temperature. Proteolytic digestion was facilitated by a combination of the enzymes Lys-C and trypsin. The initial digestion was conducted with sequencing-grade Lys-C at a ratio of 1:100 (protease:protein) for 4 h at 37 °C. Following digestion, samples were diluted with four times the initial sample volume in 50 mM ammonium bicarbonate. Subsequent digestion was performed with sequencing-grade trypsin at a ratio of 1:25 (protease:protein) overnight at 37 °C. Digestion was terminated by acidification with 2% trifluoroacetic acid (TFA) in 20% acetonitrile (ACN) (3:1 (*v*/*v*) dilution). Peptide suspensions were then purified with Pierce C18 Spin Columns from Thermo Fisher Scientific (Dublin, Ireland) and the resulting peptide samples were dried through vacuum centrifugation and re-suspended in loading buffer consisting of 2% ACN and 0.05% TFA in LC-MS grade water [63]. Samples were vortexed and sonicated to ensure an even suspension of peptides, and were stored at −80 °C prior to mass spectrometric analysis.

### 2.5. Label-Free Liquid Chromatography Mass Spectrometric Analysis

As previously described in detail [64], nano-LC-MS/MS analysis was conducted using an Ultimate 3000 NanoLC system (Dionex Corporation, Sunnyvale, CA, USA) coupled to a Q-Exactive mass spectrometer from Thermo Fisher Scientific (Dublin, Ireland). Peptide mixtures (3 µL, with a total protein amount of 0.72 µg) were loaded by an auto-sampler onto a C18 trap column (C18 PepMap, 300 µm id *×* 5 mm, 5 µm particle size, 100 Å pore size; Thermo Fisher Scientific). The trap column was switched on-line with an analytical Biobasic C18 Picofrit column (C18 PepMap, 75 µm id *×* 50 cm, 2 µm particle size, 100 Å pore size; Dionex Corporation). Peptides derived from muscle protein digestion were eluted using the following gradient (solvent B: 80% (*v*/*v*) ACN and 0.1% (*v*/*v*) formic acid in LC-MS grade water): 0% solvent B for 10.5 min, 2% solvent B for 110 min, 40% solvent B for 2.5 min, 90% solvent B for 9 min, and 2% solvent B for 43 min. The column flow rate was set to 0.25 µL/min. Data were acquired with Xcalibur software (Thermo Fisher Scientific, Dublin, Ireland, version 3.0 of Xcalibur software). The mass spectrometer was operated in positive, data-dependent mode and was externally calibrated. Survey MS scans were conducted in the mass spectrometer in the 300–1700 *m*/*z* range with a resolution of 140,000 (*m*/*z* 200) and lock mass set to 445.12003 u. Collision-induced dissociation (CID) fragmentation was carried out with the fifteen most intense ions per scan and at a resolution of 17,500. A dynamic exclusion window was applied within 30 s. An isolation window of 2 *m*/*z* and one microscan were used to collect suitable tandem mass spectra.

### 2.6. Quantitative Proteomic Profiling by Label-Free LC-MS/MS Analysis

Processing of the raw data generated from LC-MS/MS analysis was achieved using Progenesis QI for Proteomics software (version 3.1; Non-Linear Dynamics, a Waters company, Newcastle upon Tyne, UK). Firstly the data was aligned based on the LC retention time of each sample [65]. This allows for any drift in retention time, thus giving an adjusted time for all runs in the analysis. The sample run with the greatest number of peptide ions was selected as a reference run, to which the retention times of all other runs were aligned, and peak intensities were normalized [60]. Prior to exporting the MS/MS data files to Proteome Discoverer 1.4 (Thermo Fisher Scientific, Dublin, Ireland); the data was filtered using the following criteria: (i) peptide features with ANOVA ≤ 0.05 between experimental groups; (ii) mass peaks with charge states from +1 to +5; and (iii) greater than one isotope per peptide. A PepXML generic file was generated from all exported MS/MS spectra from Progenesis software (version 3.1; Non-Linear Dynamics, a Waters company, Newcastle upon Tyne, UK). This file was used for peptide identification using Proteome Discoverer 1.4 against Mascot (version 2.3, Matrix Science, Boston, MA, USA) and Sequest HT (SEQUEST HT algorithm, licence Thermo Scientific, registered trademark University of Washington, Seattle, WA, USA) and searched against the UniProtKB-SwissProt database (taxonomy: *Mus musculus*; http://www.uniprot.org/proteomes/UP000000589). A number of search parameters were used for protein identification: (i) peptide mass tolerance set to 10 ppm; (ii) MS/MS mass tolerance set to 0.02 Da; (iii) allowance of up to two missed cleavages; (iv) carbamidomethylation set as a fixed modification; and (v) methionine oxidation set as a variable modification [66]. For re-importation back into Progenesis LC-MS software for further analysis, only peptides with either ion scores of 40.00 or more (from Mascot) and peptides with XCorr scores >1.9 for singly-charged ions, >2.2 for doubly-charged ions, and >3.75 for triply-charged ions or more (from Sequest HT) were selected. Crucially, the following criteria were applied to assign a muscle-associated protein as properly identified: (i) an ANOVA score between experimental groups of ≤0.05; and (ii) proteins with ≥2 unique peptides matched and a MASCOT score ≥40. Standard bioinformatics software programs were used to group proteins based on their protein class and to identify potential protein interactions. These analyses were performed on the MS-identified proteins with a changed abundance in *mdx-4cv* fractions. This bioinformatics analysis was performed with the PANTHER database of protein families [67,68] (http://pantherdb.org/) and the STRING database of known and putative protein interactions that include both direct physical and indirect functional protein associations [69,70] (http://string-db.org/).

### 2.7. Independent Verification of Key Proteomic Hits by Comparative Immunoblot Analysis

In order to verify alterations in protein expression in crude extracts from dystrophic skeletal muscles, as identified by label-free mass spectrometry, immunoblotting of select muscle proteins was carried out per standard procedure [49]. Electrophoretic separation of proteins was achieved using standard 10% polyacrylamide gels, followed by wet transfer at 100 V for 70 min at 4 °C to Whatman Protan nitrocellulose sheets in a Transblot Cell from Bio-Rad Laboratories (Hemel-Hempstead). To minimize non-specific antibody binding, membranes were blocked for 1 h at room temperature with a milk protein solution (2.5% (*w*/*v*) fat-free milk powder in 10% phosphate-buffered saline). Nitrocellulose sheets were incubated with sufficiently-diluted primary antibodies overnight at 4 °C with gentle agitation. Membranes were subsequently washed twice with the milk protein solution for 10 min each time, followed by incubation for 1.5 h with peroxidase-conjugated secondary antibodies, diluted in the blocking buffer [60]. Further washing steps with the milk protein solution and 10% phosphate-buffered saline were carried out prior to visualization of antibody-labeled protein bands using enhanced chemiluminescence as per manufacturer’s guidelines. Densitometric scanning and statistical analysis of immunoblots were performed with a HP PSC-2355 scanner and ImageJ software (NIH, Bethesda, MD, USA; version 1.42), in conjunction with GraphPad Prism software (Graphpad Prism, San Diego, CA, USA, version 5.00), in which a *p* value < 0.05 was deemed to be statistically significant. Silver staining of protein gels and antibody labeling of the protein product of the house keeping gene that encodes lactate dehydrogenase were employed as loading controls for comparative immunoblot analyses.

### 2.8. Immunofluorescence Microscopy

In order to correlate the severe reduction of dystrophin (as a dystrophic biomarker) with the concomitant increase of collagen (as a myofibrosis biomarker), as revealed here by the mass spectrometric survey of crude *mdx-4cv* hind limb extracts, an immunofluorescence microscopical analysis of the *gastrocnemius* muscle was carried out. Freshly dissected muscles from six-month-old *mdx-4cv* mice and control C57BL/6 mice were quick-frozen in liquid nitrogen-cooled isopentane and 10 µm sections cut in a cryostat [27]. For routine immuno-labeling, tissue sections were fixed in a 1:1 (*v*/*v*) mixture of methanol and acetone for 10 min at room temperature. For dystrophin immuno-staining, unfixed cryosections were boiled in phosphate-buffered saline for 5 min as previously described in detail [71]. Tissue sections were permeabilized in 0.1% (*v*/*v*) Triton X-100 for 10 min and then blocked with 1:20 diluted normal goat serum for 30 min at room temperature. Primary antibodies to dystrophin and collagen were diluted 1:20 in phosphate-buffered saline for overnight incubation at 4 °C. Specimens were carefully washed and then incubated with fluorescently-labeled secondary antibodies, using either 1:200 diluted anti-rabbit Alexa Fluor 488 antibody or 1:200 diluted anti-mouse RRX antibody for 45 min at room temperature. Nuclei were counter-stained with 1 μg/mL bis-benzimide Hoechst 33342. Antibody-labeled tissue sections were embedded in Fluoromount G medium and viewed under a Zeiss Axioskop 2 epifluorescence microscope equipped with a digital Zeiss AxioCam HRc camera (Carl Zeiss Jena GmbH, Jena, Germany).

## 3. Results and Discussion

Although X-linked muscular dystrophy is a primary muscle disease of the membrane cytoskeleton [72], *i.e.*, the almost complete loss of the full-length Dp427 isoform of dystrophin is the underlying pathobiochemical defect that triggers progressive fiber degeneration [73], a crucial secondary alteration in dystrophinopathy is presented by extensive myofibrosis [74]. Thus, in addition to central nucleation, rounded fibers, altered myofiber sizes, inflammation, fiber branching, necrosis, and fatty deposition [75], the progressive accumulation of extracellular matrix components plays a major role in contractile weakness [76]. Due to a hyperactive connective tissue, collagens and associated proteins of the extracellular matrix accumulate in dystrophic muscles causing tissue scarring and the loss of skeletal muscle elasticity [77]. Biochemical and cell biological studies have clearly shown an increased abundance of a variety of proteoglycans and other components of the matrisome in dystrophic fibers [78,79,80,81]. Clinical investigations have established fibrosis as a pathological hallmark of dystrophinopathies [82], whereby the systematic evaluation of myofibrosis in a 10-year longitudinal study with a large number of Duchenne patients suggests that endomysial fibrosis correlates significantly with weakened muscle performance [83]. This makes changes in the extracellular matrix surrounding dystrophic fibers a robust myopathological characteristic of X-linked muscular dystrophy. In analogy to the fact that muscle-derived fibroblasts from Duchenne patients show signs of a pro-fibrotic phenotype and an enhanced proliferation rate coupled to the increased production of collagens [84,85], fibrotic changes are also observed in animal models of muscular dystrophy [86,87,88,89]. Since fibrosis is intrinsically involved in the molecular pathogenesis of muscular dystrophy, systematic studies of global protein changes in dystrophic fibers should be able to correlate the deficiency in dystrophin with altered expression levels of key components of the extracellular matrix [37].

However, a variety of mass spectrometry-based proteomic surveys of crude tissue extracts from dystrophic skeletal muscles have so far failed to properly identify the full-length dystrophin isoform and its tightly associated glycoproteins, but have exclusively shown secondary changes down-stream of the primary abnormality in Dp427 [21]. Here, we could overcome these bioanalytical issues and identify dystrophin in total muscle extracts by label-free mass spectrometry and simultaneously analyze increased levels of the fibrosis biomarker collagen. Hence, this study enabled the concurrent and systematic determination of the primary deficiency in dystrophin and its secondary effects on the entire skeletal muscle protein complement. The mass spectrometry-based proteomic classification of the assessable protein repertoire from total mouse skeletal muscle extracts resulted in the identification of 851 distinct protein species (not shown). The systematic comparison of total tissue extracts from wild type *versus mdx-4cv* hind limb muscles by label-free LC-MS/MS analysis established a change in a considerable number of these proteomically catalogued elements, *i.e.*, an altered abundance in 197 muscle-associated proteins in the dystrophic phenotype (Table 1 and Table 2). A decreased concentration was established for 43 proteins and 154 proteins exhibited a significantly increased expression level.

**Table 1:** List of identified proteins that exhibit a significantly reduced concentration in crude *mdx-4cv* hind limb muscle preparations as revealed by label-free LC-MS/MS analysis.

Accession No.	Protein Name	Unique Peptides	Confidence Score	Anova (p)	Fold Change
P11531	Dystrophin Dp427	9	457.7	0.000053	−14.61
Q8VCT4	Carboxylesterase 1D	4	175.0	0.001943	−5.85
O55137	Acyl-coenzyme A thioesterase 1	2	139.31	0.002283	−4.35
P19096	Fatty acid synthase	3	198.37	0.006235	−3.99
Q9JJW5	Myozenin-2	2	67.3	0.016575	−3.98
Q8C0M9	Isoaspartyl peptidase/L-asparaginase	3	202.6	0.000870	−3.97
P97823	Acyl-protein thioesterase 1	2	115.7	0.017276	−3.96
P32848	Parvalbumin alpha	4	262.0	0.040028	−3.94
Q61234	Alpha-1-syntrophin	3	101.6	0.000873	−3.68
P51667	Myosin regulatory light chain MLC2, slow	6	319.1	0.029470	−3.64
Q08642	Protein-arginine deiminase type-2	10	498.3	0.003334	−3.55
P09542	Myosin light chain MLC3	8	548.5	0.016031	−3.50
P16015	Carbonic anhydrase CA3	21	1652.1	0.001537	−3.43
Q8QZS1	3-hydroxyisobutyryl-CoA hydrolase, mitochondrial	3	202.7	0.002445	−3.14
Q64105	Sepiapterin reductase	7	515.0	0.002035	−3.14
P70695	Fructose-1,6-bisphosphatase isozyme 2	9	592.7	0.003223	−3.09
Q9DAK9	14 kDa phosphohistidine phosphatase	2	208.6	0.000263	−3.01
P04247	Myoglobin	16	1768.3	0.000652	−2.93
Q8BVI4	Dihydropteridine reductase	5	336.0	0.000315	−2.91
Q9DBB8	Trans-1,2-dihydrobenzene-1,2-diol dehydrogenase	2	119.5	0.026280	−2.90
Q9D358	Low molecular weight phosphotyrosine protein phosphatase	2	115.6	0.002473	−2.89
P06801	NADP-dependent malic enzyme	8	348.2	0.005720	−2.84
P21107	Tropomyosin alpha-3 chain	3	113.6	0.024475	−2.71
O55103	Periaxin	2	50.9	0.014583	−2.66
Q8R1G2	Carboxymethylenebutenolidase homolog	4	418.4	0.005828	−2.64
Q9D0K2	Succinyl-CoA:3-ketoacid coenzyme A transferase 1, mitochondrial	2	143.4	0.009917	−2.60
Q9WUZ5	Troponin I, slow skeletal muscle	2	114.0	0.033414	−2.50
P56375	Acylphosphatase-2	3	227.8	0.000551	−2.39
P17563	Selenium-binding protein 1	7	398.9	0.000733	−2.35
P14152	Malate dehydrogenase, cytoplasmic	8	588.6	0.000441	−2.33
Q9CRB9	Coiled-coil-helix-coiled-coil-helix domain-containing protein 3, mitochondrial	2	191.9	0.008307	−2.28
P70349	Histidine triad nucleotide-binding protein 1	6	504.1	0.004703	−2.27
P11404	Fatty acid-binding protein, FABP3, heart	5	407.2	0.011413	−2.21
Q01768	Nucleoside diphosphate kinase B	3	216.1	0.029418	−2.21
Q9D0S9	Histidine triad nucleotide-binding protein 2, mitochondrial	2	88.7	0.007985	−2.14
Q9CQR4	Acyl-coenzyme A thioesterase 13	2	155.3	0.009677	−2.14
P15626	Glutathione S-transferase Mu 2	5	300.4	0.019473	−2.13
P08228	Superoxide dismutase [Cu-Zn]	5	205.9	0.004575	−2.08
Q8BZA9	Fructose-2,6-bisphosphatase (TIGAR)	2	106.5	0.003999	−2.08
Q91ZJ5	UTP-glucose-1-phosphate uridylyltransferase	15	949.2	0.002697	−2.08
P63017	Heat shock cognate 71 kDa	4	465.8	0.003124	−2.03
P15327	Bisphosphoglycerate mutase	3	213.3	0.013366	−2.02
Q60864	Stress-induced-phosphoprotein 1	3	175.8	0.001854	−2.01

**Table 2:** List of identified proteins that exhibit a significantly increased concentration in crude *mdx-4cv* hind limb muscle preparations as revealed by label-free LC-MS/MS analysis.

Accession No.	Protein Name	Unique Peptides	Confidence Score	Anova (p)	Fold Change
Q61879	Myosin-10	2	89.2	0.000004	*mdx* only
P11276	Fibronectin	2	133.4	0.002667	271.95
Q00898	Alpha-1-antitrypsin 1–5	5	571.1	0.000004	187.00
Q02788	Collagen alpha-2(VI) chain	2	102.6	0.000993	44.37
P28653	Biglycan	4	232.4	0.004166	17.82
Q8R5J9	PRA1 family protein 3	2	45.7	0.000212	16.11
Q9ESD7	Dysferlin	2	62.5	0.004132	16.08
Q8VDD5	Myosin-9	4	267.7	0.009126	15.75
Q9D154	Leukocyte elastase inhibitor A	9	608.0	0.000120	12.58
P09541	Myosin light chain MLC4	7	467.0	0.000000	12.04
Q8VCM7	Fibrinogen gamma chain	3	75.5	0.000341	11.00
Q8K0E8	Fibrinogen beta chain	11	586.9	0.000203	10.96
P62835	Ras-related protein Rap-1A	2	111.6	0.004823	10.62
P99024	Tubulin beta-5 chain	4	211.9	0.001015	9.81
Q3TMP8	Trimeric intracellular cation channel type A	2	192.7	0.004054	8.93
P21981	Protein-glutamine gamma-glutamyltransferase 2	2	98.6	0.023285	8.77
O89053	Coronin-1A	2	83.2	0.004520	8.09
P51881	ADP/ATP translocase 2	2	56.4	0.004098	8.06
P97449	Aminopeptidase N	2	60.6	0.002562	7.79
Q9D1G3	Protein-cysteine *N*-palmitoyltransferase HHAT-like protein	5	363.5	0.009730	6.54
P28665	Murinoglobulin-1	15	759.7	0.002456	6.50
P03921	NADH-ubiquinone oxidoreductase chain 5	3	200.0	0.018874	6.27
Q8VDN2	Sodium/potassium-transporting ATPase subunit alpha-1	5	274.2	0.015103	6.07
P68433	Histone H3.1	3	261.4	0.011746	5.98
P11087	Collagen alpha-1(I) chain	4	205.2	0.016481	5.91
Q99JY9	Actin-related protein 3	3	152.9	0.000084	5.58
Q7TSH2	Phosphorylase b kinase regulatory subunit beta	2	72.7	0.035782	5.49
Q99MQ4	Asporin	5	354.1	0.000546	5.42
Q61233	Plastin-2	6	251.2	0.000108	5.38
P41216	Long-chain-fatty-acid-CoA ligase 1	5	242.7	0.020046	5.19
Q91V79	Fat storage-inducing transmembrane protein 1	2	114.1	0.012321	5.15
Q6PIE5	Sodium/potassium-transporting ATPase subunit alpha-2	6	279.1	0.010979	5.02
P10107	Annexin A1	5	349.4	0.000751	4.98
Q68FD5	Clathrin heavy chain 1	10	600.2	0.000416	4.81
Q00623	Apolipoprotein A-I	14	959.8	0.000386	4.63
A2AMM0	Muscle-related coiled-coil protein	4	173.6	0.000173	4.60
Q9CR62	Mitochondrial 2-oxoglutarate/malate carrier protein	2	61.5	0.032119	4.60
P14094	Sodium/potassium-transporting ATPase subunit beta-1	2	99.2	0.010749	4.38
Q9DBG6	Dolichyl-diphosphooligosaccharide-protein glycosyltransferase subunit 2	2	123.2	0.017838	4.26
P13020	Gelsolin	10	696.4	0.000002	4.17
P62908	40S ribosomal protein S3	3	191.5	0.015578	4.15
Q6ZWV3	60S ribosomal protein L10	2	130.3	0.035667	4.11
Q01339	Beta-2-glycoprotein 1	3	76.3	0.016181	4.11
Q61147	Ceruloplasmin	2	104.5	0.005975	4.08
Q60854	Serpin B6	13	834.5	0.000105	3.99
Q99P72	Reticulon-4	2	145.7	0.007711	3.95
Q8BH59	Calcium-binding mitochondrial carrier protein Aralar1	7	511.8	0.014147	3.91
P20918	Plasminogen	2	160.2	0.018437	3.88
O09161	Calsequestrin-2	3	162.3	0.000414	3.87
Q61838	Alpha-2-macroglobulin	27	1386.6	0.004622	3.78
P16546	Spectrin alpha chain, non-erythrocytic 1	5	348.1	0.006923	3.74
O89104	Synaptophysin-like protein 2	2	236.0	0.009844	3.73
E9PZQ0	Ryanodine receptor 1	27	1821.4	0.009260	3.71
Q71LX4	Talin-2	2	46.7	0.024488	3.69
P68369	Tubulin alpha-1A chain	12	719.0	0.006406	3.67
P22752	Histone H2A type 1	4	199.0	0.001916	3.67
P29621	Serine protease inhibitor A3C	2	240.9	0.002558	3.66
P01872	Ig mu chain C region	3	242.2	0.004580	3.65
Q9EQK5	Major vault protein	2	108.4	0.002870	3.58
P07356	Annexin A2	9	662.8	0.000916	3.55
P00405	Cytochrome c oxidase subunit 2	3	118.8	0.015387	3.52
P22599	Alpha-1-antitrypsin 1-2	3	186.1	0.004003	3.46
Q6ZWY9	Histone H2B type 1-C/E/G	3	310.5	0.006482	3.46
P26039	Talin-1	3	199.2	0.001177	3.36
P18826	Phosphorylase b kinase regulatory subunit alpha, skeletal muscle isoform	3	235.7	0.008094	3.34
P15864	Histone H1.2	2	94.9	0.011066	3.28
P62806	Histone H4	5	312.4	0.010666	3.28
Q8BMS1	Trifunctional enzyme subunit alpha, mitochondrial	9	762.3	0.011513	3.25
P26041	Moesin	5	180.3	0.000563	3.23
P14148	60S ribosomal protein L7	3	181.6	0.013313	3.22
Q8VEM8	Phosphate carrier protein, mitochondrial	5	264.8	0.014880	3.22
Q61207	Sulfated glycoprotein 1	3	122.3	0.003193	3.13
Q04857	Collagen alpha-1(VI) chain	3	87.7	0.018019	3.12
P61027	Ras-related protein Rab-10	3	118.3	0.001577	3.12
P28654	Decorin	5	350.6	0.016544	3.10
Q9D783	Kelch-like protein 40	6	330.1	0.001034	3.09
P97927	Laminin subunit alpha-4	3	144.0	0.002278	3.03
O08532	Voltage-dependent calcium channel subunit alpha-2/delta-1	5	289.9	0.011742	3.03
Q8BTM8	Filamin-A	3	123.0	0.006675	2.98
A2AUC9	Kelch-like protein 41	12	774.9	0.008240	2.96
P07758	Alpha-1-antitrypsin 1-1	9	691.9	0.014277	2.93
Q9CVB6	Actin-related protein 2/3 complex subunit 2	3	154.1	0.000307	2.91
P18760	Cofilin-1	5	305.0	0.004511	2.90
Q8CI43	Myosin light chain 6B	2	66.6	0.007575	2.89
P48962	ADP/ATP translocase 1	9	579.2	0.017242	2.85
Q9D6F9	Tubulin beta-4A chain	5	478.9	0.003730	2.78
Q8R429	Sarcoplasmic/endoplasmic reticulum calcium ATPase 1	39	3489.2	0.019926	2.77
P09405	Nucleolin	2	453.6	0.005112	2.75
P24369	Peptidyl-prolyl cis-trans isomerase B	2	132.9	0.000596	2.72
P13541	Myosin-3	5	425.1	0.018031	2.71
P48036	Annexin A5	8	459.7	0.003110	2.70
P29391	Ferritin light chain 1	9	652.0	0.005573	2.68
P62874	Guanine nucleotide-binding protein G(I)/G(S)/G(T) subunit beta-1	3	178.5	0.001344	2.67
P32261	Antithrombin-III	3	178.1	0.004703	2.65
P07759	Serine protease inhibitor A3K	9	716.8	0.002934	2.64
Q7TMM9	Tubulin beta-2A chain	10	632.2	0.001380	2.64
P47911	60S ribosomal protein L6	2	145.7	0.011238	2.59
Q91X72	Hemopexin	13	636.3	0.016945	2.58
P14869	60S acidic ribosomal protein	3	86.0	0.004999	2.56
Q9JK53	Prolargin	5	227.2	0.005281	2.56
P48678	Prelamin-A/C	25	1641.1	0.000349	2.55
Q9CQQ7	ATP synthase F(0) complex subunit B1, mitochondrial	5	211.7	0.003672	2.54
Q9CZM2	60S ribosomal protein L15	2	104.9	0.024828	2.51
P62962	Profilin-1	4	270.9	0.015520	2.49
Q91VI7	Ribonuclease inhibitor	7	339.1	0.013370	2.48
Q07076	Annexin A7	2	120.8	0.001600	2.46
Q8R4E4	Myozenin-3	2	100.5	0.001180	2.45
P62141	Serine/threonine-protein phosphatase PP1-beta catalytic subunit	2	177.1	0.001508	2.45
Q70IV5	Synemin	2	104.4	0.000186	2.43
P20152	Vimentin	15	1088.5	0.002258	2.42
P35980	60S ribosomal protein L18	3	154.7	0.018170	2.41
Q62000	Mimecan	7	497.7	0.016065	2.41
Q60930	Voltage-dependent anion-selective channel protein 2	9	649.6	0.040913	2.36
Q8BFR5	Elongation factor Tu, mitochondrial	9	509.1	0.004815	2.36
Q9WTR5	Cadherin-13	3	245.5	0.006036	2.36
P47757	*F*-actin-capping protein subunit beta	2	143.1	0.003431	2.34
Q9Z1E4	Glycogen [starch] synthase, muscle	4	199.6	0.019418	2.32
P97384	Annexin A11	5	201.6	0.000422	2.32
P68040	Guanine nucleotide-binding protein subunit beta-2-like 1	6	311.8	0.000693	2.31
Q91WD5	NADH dehydrogenase [ubiquinone] iron-sulfur protein 2, mitochondrial	8	305.4	0.001414	2.31
P47963	60S ribosomal protein L13	2	109.2	0.023095	2.30
P50543	Protein S100-A11	2	136.8	0.003349	2.30
Q61425	Hydroxyacyl-coenzyme A dehydrogenase, mitochondrial	2	115.4	0.012822	2.29
P05213	Tubulin alpha-1B chain	2	209.9	0.016043	2.29
P68368	Tubulin alpha-4A chain	3	305.2	0.006357	2.28
Q8BG05	Heterogeneous nuclear ribonucleoprotein A3	2	82.4	0.040172	2.28
Q6ZWX6	Eukaryotic translation initiation factor 2 subunit 1	2	73.2	0.018602	2.27
A2AAJ9	Obscurin	9	474.2	0.004426	2.26
P62889	60S ribosomal protein L30	4	279.4	0.001887	2.26
Q9DC69	NADH dehydrogenase 1 alpha subcomplex subunit 9, mitochondrial	3	184.7	0.014362	2.26
P50544	Very long-chain specific acyl-CoA dehydrogenase, mitochondrial	3	148.7	0.002723	2.25
P63101	14-3-3 protein zeta/delta	4	390.8	0.006991	2.23
Q8BK84	Dual specificity phosphatase DUPD1	2	146.8	0.004756	2.21
Q9DB20	ATP synthase subunit O, mitochondrial	4	277.8	0.010221	2.18
Q3MI48	Junctional sarcoplasmic reticulum protein 1	3	145.6	0.020961	2.17
P14824	Annexin A6	5	539.1	0.003047	2.15
Q03265	ATP synthase subunit alpha, mitochondrial	11	778.3	0.001158	2.10
Q9JJZ2	Tubulin alpha-8 chain	2	68.2	0.023642	2.10
Q60605	Myosin light polypeptide 6	4	300.2	0.010440	2.09
Q99JB8	Protein kinase C and casein kinase II substrate protein 3	8	439.7	1.20E-05	2.09
P31001	Desmin	18	1622.8	0.001451	2.09
Q61292	Laminin subunit beta-2	5	233.6	0.013369	2.08
P40124	Adenylyl cyclase-associated protein 1	2	52.1	0.001465	2.07
Q8VHX6	Filamin-C	20	1257.4	0.038298	2.07
P51885	Lumican	5	346.1	0.021066	2.07
Q9CZU6	Citrate synthase, mitochondrial	5	373.2	0.014760	2.07
O35129	Prohibitin-2	3	122.3	0.004054	2.06
Q9DB60	Prostamide/prostaglandin F synthase	2	48.5	0.001144	2.05
O88342	WD repeat-containing protein 1	3	205.6	0.004625	2.04
P14602	Heat shock protein beta-1	6	349.6	0.002016	2.04
P14733	Lamin-B1	5	244.4	0.001897	2.03
Q60936	Chaperone activity of bc1 complex-like, mitochondrial	2	98.4	0.028276	2.02
P35979	60S ribosomal protein L12	3	172.6	0.020128	2.02
Q02053	Ubiquitin-like modifier-activating enzyme 1	2	170.9	0.030810	2.01

### 3.1. Label-Free LC-MS/MS Analysis of Decreased Proteins in Total mdx-4cv Muscle Extracts

The most crucial aspect of this report is the mass spectrometric classification of dystrophin (P11531) in whole tissue preparations using comparative proteomics, and the unequivocal identification of this membrane cytoskeletal component as the most significantly reduced protein in crude *mdx-4cv* skeletal muscle extracts (Table 1). This is a considerable bioanalytical achievement in relation to the biochemical identification of a low-abundance and membrane-associated muscle protein within total tissue homogenates [90]. The skeletal muscle proteome has been extensively catalogued and characterized in its fiber type specific composition by mass spectrometry-based proteomics [91,92,93,94,95]. Although individual members of the large family of dystrophin proteins ranging from approximately 45 to 427 kDa [3] have been described in large-scale proteomic surveys [95], a considerable number of investigations employing comparative proteomics of crude tissue extracts have failed to identify the dystrophin-glycoprotein complex [20]. The reduced density of dystrophin in *mdx-4cv* muscles, as shown here by label-free mass spectrometric analysis, agrees with the pathobiochemical concept that the drastic reduction in the full-length dystrophin isoform Dp427 is the primary defect in dystrophinopathies [2]. This proteomic finding also supports the results from previous studies that have shown a very low rate of revertant Dp427-positive fibers in the *mdx-4cv* mouse model of Duchenne muscular dystrophy [50,51,52,53]. In addition to Dp427, another member of the dystrophin-glycoprotein complex that exhibits a drastically reduced concentration in muscular dystrophy was identified by mass spectrometry in total extracts, *i.e*., the adapter protein α1-syntrophin of 54 kDa (Q61234). This agrees with the previous immunoblot analysis of skeletal muscles from the conventional *mdx* mouse [96] and biopsy specimens from Duchenne patients [97].

In addition, interesting muscle proteins of reduced density in dystrophin-deficient fibers, that are potentially useful for the establishment of a comprehensive biomarker signature of muscular dystrophy [98], are the Z-line protein myozenin-2 (Q9JJW5), parvalbumin (P32848), myoglobin (P04247), carbonic anhydrase CA3 (P16015), glutathione S-transferase (P15626), and fatty acid binding protein FABP3 (P11404), as listed in Table 1. These changes indicate alterations in α-actinin- and γ-filamin-binding, impaired cytosolic Ca^2+^-buffering, modified CO_2_-removal mechanisms and a changed biotransformation capacity, as well as a reduced ability for oxygen transportation and fatty acid utilization. The metabolite transporter FABP3 appears to be a limiting factor of oxidative muscle metabolism [99] and was established as a proteomic biomarker of aerobic capacity in skeletal muscles [100]. The change in FABP3 expression indicates alterations or physiological adaptations in relation to oxidative metabolism in dystrophic fibers. A reduced concentration of contractile and regulatory proteins was shown to occur in the thick filament (myosin light chains MLC2 and MLC3) and the thin filament (tropomyosin alpha-3, troponin TnI), suggesting considerable rearrangements within the contractile apparatus of dystrophic fibers [101].

A recent comparative label-free mass spectrometric analysis of mildly *versus* severely affected *mdx* skeletal muscles has revealed a reduced concentration of myoglobin in diaphragm, *soleus*, *extensor digitorum longus*, and *flexor digitorum brevis* muscle, lower levels of parvalbumin in diaphragm, *soleus*, and *flexor digitorum brevis* muscle, as well as decreases of myozenin in diaphragm, *extensor digitorum longus*, and *flexor digitorum brevis* muscle [102]. This agrees with the evaluation of the *mdx-4cv* hind limb musculature shown here and suggests that the relatively abundant proteins myoglobin, parvalbumin, and myozenin present suitable biomarker candidates for the general verification of secondary changes in muscular dystrophy. 

### 3.2. Label-Free LC-MS/MS Analysis of Increased Proteins in Total mdx-4cv Muscle Extracts

A large number of muscle-associated proteins were identified to exhibit an increased concentration in Dp427-deficient skeletal muscles. As listed in Table 2, the label-free mass spectrometric analysis of crude tissue extracts from *mdx-4cv* hind limb muscles revealed drastically elevated levels of components of the extracellular matrix and the cytoskeletal network. These proteome-wide changes suggest both a myofibrosis-related accumulation of collagens and their associated components of the matrisome [20], as well as the compensatory up-regulation of cytoskeletal structures to partially counterbalance the loss of dystrophin [21]. The statistical q-values of the proteins listed in Table 1 and Table 2 are presented in supplementary Table S1.

The most significantly increased protein was identified as myosin-10, which is defined as an unconventional non-muscle type of myosin with specialized cellular functions in cell shape provision, cytokinesis and actin cytoskeletal organization, as well as stabilization of collagen synthesis [103]. Immunoblotting with an antibody to myosin-10 did not result in sufficiently specific labeling of this protein, so this proteomic finding could not be further evaluated by Western blotting (not shown). The increased levels of myosin-9 (Q8VDD5) and myosin-10 (Q61879), also referred to as non-muscle myosin heavy chains NMMHC II-A and II-B, in dystrophic muscle agree with the idea of enhanced cellular proliferation rates and increased synthesis of components of the matrisome in muscular dystrophy [37]. Crucial extracellular matrix proteins with an increased concentration were identified as collagen alpha-1(VI) chain (Q04857), alpha-2(VI) chain (Q02788), and collagen alpha-1(I) chain (P11087), as well as fibronectin (P11276), biglycan (P28653), asporin (Q99MQ4), decorin (P28654), prolargin (Q9JK53), mimecan (Q62000), and lumican (P51885). Fibronectin is an established serum biomarker of X-linked muscular dystrophy [40] and released at high levels into the circulatory system of Duchenne patients [21]. These severe changes in the extracellular matrix strongly suggest that myofibrosis plays a central pathobiochemical role in the molecular pathogenesis of progressive muscular dystrophy [77]. Collagen type IV was previously shown to be drastically increased in diaphragm, *flexor digitorum brevis*, and *interosseus* muscles [35,102]. The up-regulated collagen type VI is a major filament-forming collagen of the interstitial matrix that closely interacts with other collagens, fibronectin, biglycan, decorin, and integrins [104]. Hence, the dystrophinopathy-related accumulation of the fibrillar extracellular matrix is clearly associated with the concomitant increase in decorin, asporin, and prolargin, as shown in this report, as well as dermatopontin and the matricellular protein periostin, as previously shown by comparative subproteomic and proteomic studies [35,36,37,49]. Subunits of fibrinogen (Q8VCM7, Q8KOE8) were also identified as being increased in *mdx-4cv* hind limb muscles, as previously shown to occur in the aged and severely fibrotic *mdx* diaphragm [35]. This supports the concept that significant myofibrotic changes alter the extracellular environment of dystrophic muscle fibers [80], since fibrinogen affects the transforming growth factor-β/alternative macrophage activation pathway in dystrophin-deficient muscles [105].

The apparent up-regulation of tubulin (Q9ERD7; Q9D6F9; P99024), vinculin (Q64727), talin (P26039, Q71LX4), and vimentin (P20152) suggests a compensatory rebalancing of the weakened cytoskeletal system in Dp427-lacking muscle fibers. The restructuring of intermediate filaments and microtubular networks may stabilize the intracellular matrix in the absence of the sarcolemmal dystrophin lattice during contractile and mechanical strains. Importantly, vimentin was recently identified as being increased in all investigated contractile tissues from the conventional *mdx* mouse, ranging from mildly to moderately to severely dystrophic skeletal muscles. This study included the comparative proteomic evaluation of *interosseus*, *flexor digitorum brevis*, *soleus*, *extensor digitorum longus*, and diaphragm muscles [102]. The increased levels of the nuclear envelope protein lamin (isoforms A/C (P48678) and B (P14733)) also agrees with the proteomic evaluation of individual *mdx* hind limb muscles and the diaphragm [102]. In skeletal muscles, isoforms of annexin are linked to the continued maintenance of the cytoskeletal network and the provision of extracellular matrix integrity [106]. This would agree with the proteomic finding that annexin isoforms A1, A2, A5, A6, A7, and A11 (P10107; P07356; P14824; P48036; Q07076; P97384), which are also majorly involved in Ca^2+^-handling, are increased in *mdx-4cv* muscles (Table 2). Annexin isoforms therefore represent universal biomarkers of muscular dystrophy, since they were also shown to be increased in diaphragm, *soleus*, *extensor digitorum longus*, *interosseus*, and *flexor digitorum brevis* muscles [102]. In addition, the presence of high levels of dysferlin (Q9ESD7) indicates the initiation of sarcolemmal repair mechanisms to counteract micro-rupturing of the deteriorated muscle surface membrane system [107]. A highly elevated protein in dystrophic muscle preparations was identified as the anti-protease molecule named alpha-1-antitrypsin (Q00898, P22599). Since alpha-1-antitrypsin is also involved in anti-inflammatory responses [108], the increased abundance of this protein might be a protective reaction to reduce the pathobiochemical impact of excessive proteolytic degradation and inflammatory damage to dystrophin-deficient fibers. In contrast to the observed increase in non-muscle myosin-9 (Q8VDD5) and myosin-10 (Q61879) and their proposed role in myofibrosis, the elevated levels of the embryonic myosin heavy chain isoforms myosin-3 (P13541) and myosin-8 (Accession No.: P13542; Confidence score: 499.8; Anova (p): 0.013850; Fold change: 8.63; identified by only 1 unique peptide, and therefore not listed in Table 2) has to be interpreted as changes downstream of the cytoskeletal damage pathway within the overall organization of the contractile apparatus. Although perinatal myosin-8 is clearly present in adult skeletal muscles [92], its drastic increase in dystrophic fibers indicates remodelling within dystrophic fibers and/or the increased recruitment of newly differentiated myofibers with a predominant embryonic protein expression pattern. A variety of conventional myosin light chains (P09541, Q8CI43, and Q60605) were shown to be increased in *mdx-4cv* hind limb muscles, agreeing with the above outlined idea that substantial rearrangements occur within the contractile apparatus of dystrophic fibers [101].

A considerable number of comparative proteomic studies of muscular dystrophy have used gel electrophoretic approaches for large-scale protein separation [20]. These investigations have usually not covered potential changes in extremely high-molecular-mass proteins and/or integral membrane proteins in a comprehensive way [90]. In this report, the application of liquid chromatography and sensitive label-free mass spectrometry has at least partially overcome this bioanalytical problem and successfully identified major metabolic and physiological players involved in the regulation of a variety of cellular processes. This included a large number of mitochondrial proteins and metabolite transporters, such as ADP/ATP translocase, NADH-ubiquinone oxidoreductase, NADH dehydrogenase, Ca^2+^-binding mitochondrial carrier protein, cytochrome c oxidase, phosphate carrier protein, ATP synthase, citrate synthase, acyl-CoA dehydrogenase, and the voltage-dependent anion-selective channel VDAC2 (Table 2). The mass spectrometric identification of extremely large muscle-associated proteins included obscurin (A2AAJ9), a giant myofibrillar protein of approximately 720 kDa [109], whose molecular dimensions would be too bulky for routine proteomic analysis by two-dimensional gel electrophoresis [90].

Importantly, the mass spectrometric analysis has identified elevated levels of major high-molecular mass proteins involved in the regulation and maintenance of the muscle membrane potential, excitation-contraction coupling and muscle relaxation, such as the Na^+^/K^+^-ATPase (Q6PIE5, P14094), the voltage-sensing dihydropyridine receptor (O08532), the ryanodine receptor (E9PZQ0) and the sarcoplasmic reticulum Ca^2+^-ATPase (Q8R429). The interplay between the l-type Ca^2+^-channel dihydropyridine receptor of the transverse tubules and the ryanodine receptor RyR1 Ca^2+^-release channel complex of the triads is responsible for the swift coupling between sarcolemmal depolarization and Ca^2+^-release to trigger muscle contraction, the Na^+^/K^+^-ATPase maintains the resting membrane potential over the sarcolemma, and the fast SERCA1 type Ca^2+^-ATPase induces muscle relaxation by the re-uptake of Ca^2+^-ions into the lumen of the sarcoplasmic reticulum [110]. All four proteins exist in high-molecular-mass complexes as integral protein assemblies and exhibit extensive hydrophobic peptide domains. The successful identification of these key physiological regulators demonstrates the improved protein coverage of label-free mass spectrometry as compared to purely gel-based comparative studies [49]. The increased concentration of the ion pumps and ion channels suggests major restructuring within the regulatory pathways of *mdx-4cv* hind limb muscles. Degenerating and fibrotic skeletal muscles seem to counteract impaired cellular signaling and ion fluxes by the up-regulation of essential voltage sensors, ion release channels and ion pumps to maintain optimum excitation-contraction coupling and stabilize the resting membrane potential over the sarcolemma in dystrophic fibers [111].

### 3.3. Distribution of Protein Changes in Dystrophic mdx-4cv Hind Limb Muscles

**Figure 1:**
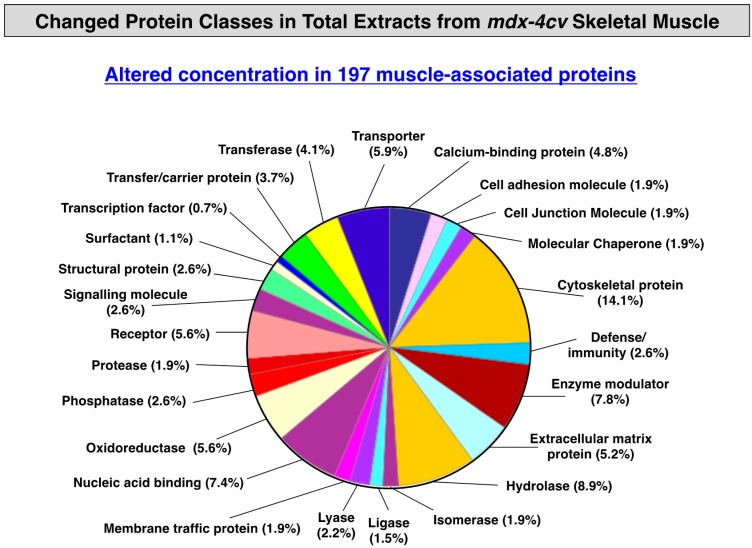
Summary of changed protein classes in total tissue extracts from *mdx-4cv* hind limb muscle. In order to identify the clustering of protein classes based on the mass spectrometric analysis of crude extracts from wild type *versus* dystrophic skeletal muscle specimens (Table 1 and Table 2), the bioinformatics software program PANTHER [67,68] was used.

To illustrate the overall distribution of variations in the expression of protein classes and potential interaction patterns between altered proteins, standard bioinformatics analyses of the mass spectrometrically-identified proteins with a changed density in *mdx-4cv* hind limb muscles were carried out. As shown in the PANTHER analysis of Figure 1 [67,68], the proteomic analysis of crude tissue extracts from dystrophic skeletal muscles revealed the highest number of alterations in the class of cytoskeletal proteins. This agrees with the biomedical fact that X-linked muscular dystrophy is a primary disorder of the membrane cytoskeleton and that the almost complete loss of dystrophin isoform Dp427 triggers massive changes in the overall cytoskeletal network within muscle fibers. This would include secondary reductions of directly and indirectly associated cytoskeletal elements, but also the compensatory up-regulation of stabilizing proteins belonging to the microtubules and intermediate filament systems (Table 1 and Table 2).

A bioinformatics STRING analysis was carried out [69,70] and the resulting interaction map of changed proteins in total muscle extracts from *mdx-4cv* muscles highlights the central position of Dp427 within the large network of altered protein species. Figures S1 and S2 in the supplementary materials show the interaction patterns between the membrane cytoskeletal change due to dystrophin deficiency and secondary effects on other major protein hubs, such as the extracellular matrix, the contractile apparatus and the intracellular matrix consisting of a variety of interconnecting cytoskeletal systems (Table 1 and Table 2). Protein changes in essential cellular processes that are involved in the excitation-contraction-relaxation cycle, physiological regulation, the uptake and transportation of metabolites, muscle energy metabolism, the extracellular matrix, the cytoskeleton and the stress response appear to majorly affect contractile functions in X-linked muscular dystrophy.

### 3.4. Verification of Proteomic Changes in Dystrophic mdx-4cv Hind Limb Muscles

**Figure 2:**
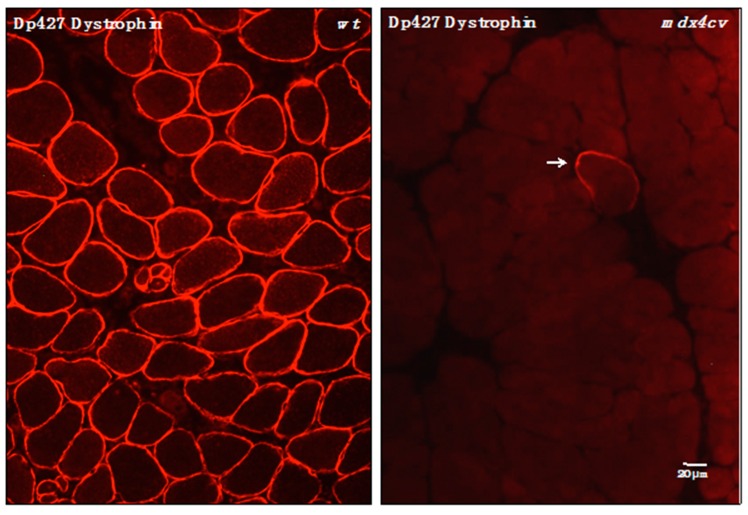
Immunofluorescence microscopical localization of dystrophin isoform Dp427 in transverse cryosections from normal wild-type (*wt*) *versus* dystrophic *mdx-4cv gastrocnemius* muscle. Shown is the labeling of the sarcolemma in normal *wt* muscle using antibodies to dystrophin [71]. In stark contrast, the Dp427 isoform is almost completely absent from *mdx-4cv* muscle tissue. The arrow indicates a dystrophin-positive revertant fiber, which is extremely rare in the *mdx-4cv* mouse model of Duchenne muscular dystrophy [50].

In order to independently verify the findings from the proteomic analysis of total tissue extracts from wild-type *versus mdx-4cv* hind limb muscles, immunofluorescence microscopy and immunoblotting was carried out. The label-free mass spectrometric analysis of crude tissue extracts from *mdx-4cv* hind limb muscles, presented here, has for the first time identified the full-length Dp427 isoform of the membrane cytoskeletal protein dystrophin in a comparative proteomic study. In contrast to previous mass spectrometric surveys of normal *versus* dystrophic tissue preparations [20,21], that have not used pre-fractionation methodology or immuno precipitation approaches, this report has enabled the simultaneous evaluation of dystrophin deficiency and secondary downstream changes in the muscle proteome. Figure 2 clearly demonstrates that the *mdx-4cv* mouse model of X-linked muscular dystrophy exhibits only very few dystrophin-positive revertant fibers [50]. This agrees with the mass spectrometric data presented in Table 1 and the well-established pathobiochemical finding that the almost complete absence of dystrophin is the underlying primary defect in dystrophinopathies [3,72].

**Figure 3:**
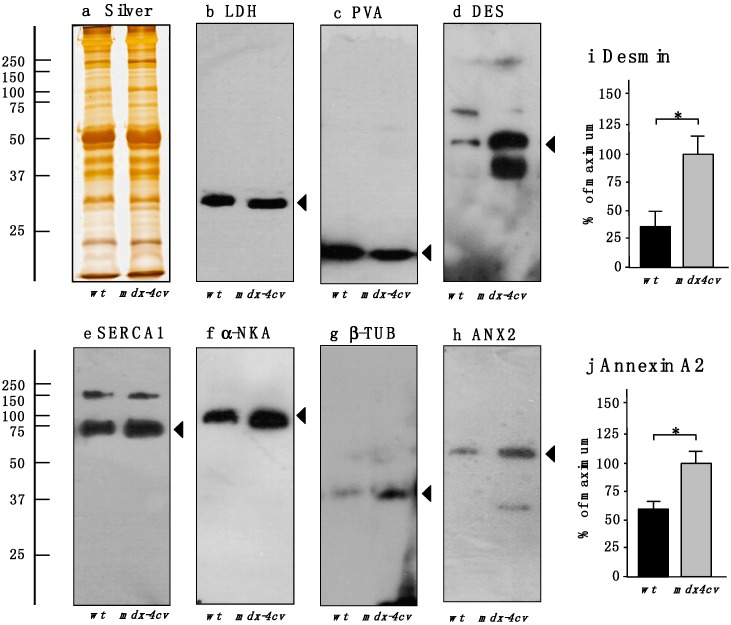
Comparative immunoblot analysis of normal wild-type (*wt*) *versus* dystrophic *mdx-4cv* hind limb skeletal muscles. Shown is a representative silver-stained gel (**a**) and immunoblots (**b**–**h**). Lanes one and two represent total extracts from control *wt* muscle and dystrophic *mdx-4cv* skeletal muscle, respectively. Blots were labeled with antibodies to lactate dehydrogenase (LDH) (**b**); the cytosolic Ca^2+^-binding protein parvalbumin (PVA) (**c**); the intermediate filament protein desmin (DES) (**d**); the fast SERCA1 isoform of the sarcoplasmic reticulum Ca^2+^-ATPase (**e**) the α-subunit of the sarcolemmal Na^+^/K^+^-ATPase (α-NKA) (**f**); the microtubular protein β-tubulin (β-TUB) (**g**) and annexin isoform ANX2 (**h**). Arrowheads mark the main immuno-labeled protein bands in individual panels. Graphical representations of the immuno-decoration levels for desmin and annexin in normal *versus mdx-4cv* skeletal muscles are shown in panels (**i**,**j**): Student’s *t*-test, unpaired; *n* = 4; * *p* < 0.05.

The immunoblotting survey shown in Figure 3 outlines the comparative analysis of a select number of key proteomic hits listed in Table 1 and Table 2. For loading controls, a silver-stained protein gel and the unchanged abundance of lactate dehydrogenase is presented in Figure 3a,b. The drastic reduction in the cytosolic Ca^2+^-binding protein parvalbumin was confirmed by immunoblotting (Figure 3c), as well as the general trend of an increased concentration of the intermediate filament protein desmin, the sarcoplasmic reticulum SERCA1 Ca^2+^-ATPase, the sarcolemmal Na^+^/K^+^-ATPase, the microtubulular protein β-tubulin and annexin isoform ANX2 (Figure 3d–h). The statistical evaluation of the increased abundance of desmin and annexin is shown in Figure 3i,j. These findings are in agreement with the proteomic data presented in this report and verify that X-linked muscular dystrophy is associated with impaired ion homeostasis in the cytosol and luminal compartments in muscle fibers, and the compensatory up-regulation of regulatory and stabilizing proteins of the cytoskeletal network.

**Figure 4:**
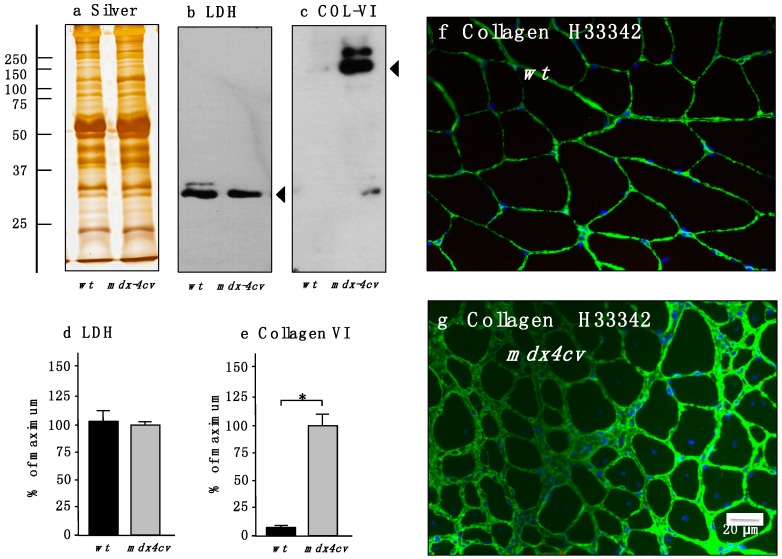
Immunoblot analysis and immunofluorescence microscopical localization of collagen in normal *versus* dystrophic *mdx-4cv gastrocnemius* muscle. Shown is a representative silver-stained gel (**a**) and immunoblots (**b**,**c**). Lanes one and two represent total extracts from control *wt* muscle and dystrophic *mdx-4cv* skeletal muscle, respectively. Blots were labeled with antibodies to lactate dehydrogenase (LDH) (**b**) and collagen isoform COL-VI (**c**). Arrowheads mark the main immuno-labeled protein bands in individual panels. Graphical representations of the immuno-decoration levels for lactate dehydrogenase and collagen in normal *versus mdx-4cv* skeletal muscles are shown in panels (**d**,**e**): Student’s *t*-test, unpaired; *n* = 4; * *p* < 0.05. The immunofluorescence microscopy panels (**f**,**g**) show the labeling of the extracellular matrix in normal *wt versus mdx gastrocnemius* muscle, respectively, using antibodies to collagen COL-VI. In Dp427-deficient *mdx-4cv* muscle tissue the levels of collagen are greatly increased. Nuclei were stained with the DNA binding dye bis-benzimide Hoechst 33342 (H33342).

Since myofibrosis is an important clinical feature of Duchenne muscular dystrophy [77] and in order to correlate the loss of dystrophin with the dramatic increase in collagen, as demonstrated here by the label-free mass spectrometric analysis of total *mdx-4cv* skeletal muscle extracts, the molecular fate of collagen isoform COL-VI was evaluated by immunoblotting and immunofluorescence microscopy. Figure 4 clearly demonstrates an increased abundance of this extracellular matrix protein. In contrast to comparable levels of overall protein and lactate dehydrogenase (Figure 4a,b,d), the concentration of collagen is significantly elevated in Dp427-deficient muscle preparations (Figure 4c,e). Immunofluorescence microscopy agreed with this finding and showed considerably increased labeling of collagen in the interstitial space of muscle fibers in transverse cryosections (Figure 4f,g).

## 4. Conclusions

Although Duchenne muscular dystrophy is a monogenic disorder of the neuromuscular system with defined genetic abnormalities and distinct clinical features, the secondary pathobiochemical changes due to the primary loss of the membrane cytoskeletal protein dystrophin are extremely complex. In the past, comparative proteomic studies using total muscle tissue extracts have greatly helped to improve our general understanding of the molecular pathogenesis of dystrophinopathies, but have mostly focused on alterations downstream of the dystrophin-glycoprotein complex. Here, we have tried to address this issue in the field of muscular dystrophy research by employing sensitive label-free mass spectrometry for the unequivocal identification of the full-length Dp427 isoform of dystrophin in total tissue extracts from hind limb homogenates in a comparative study. Dystrophin was found to be the most significantly reduced protein species in the *mdx-4cv* animal model of dystrophinopathy. This agrees with the very low rate of dystrophin-positive revertant fibers in *mdx-4cv* skeletal muscles, as confirmed by immunofluorescence microscopy. Since dystrophin was identified by mass spectrometry, the reduction in the Dp427 isoform could then be directly related to secondary changes in other muscle protein families. Muscular dystrophy was shown to be associated with alterations in contractile proteins, molecular chaperones, metabolite transporters, cell signaling proteins, ion handling proteins, and a variety of enzymes. The identified proteome-wide disturbances reflect the high degree of cellular stress, physiological impairments, and metabolic changes in muscular dystrophy, as well as potential compensatory mechanisms to maintain cytoskeletal stability in Dp427-deficient muscle fibers. Importantly, the simultaneous pathoproteomic evaluation of dystrophin and downstream changes in the *mdx-4cv* mouse model of Duchenne muscular dystrophy revealed considerable increases in markers of myofibrosis, such as collagens, fibronectin, biglycan, asporin, decorin, prolargin, mimecan, and lumican. This is a crucial proteomic finding, since the progressive accumulation of collagen and associated fibrotic changes directly correlate with the loss in motor function in Duchenne patients. This makes certain collagen isoforms and other proteins of the matrisome excellent biomarker candidates for the improved diagnostic and prognostic evaluation of muscular dystrophy-related myofibrosis. A select group of altered muscle proteins, as identified by label-free mass spectrometry in this report, could form the scientific basis for establishing an improved list of robust and muscle-associated biomarkers of dystrophinopathy. These new indicators of secondary changes in muscular dystrophy may be especially useful for the objective monitoring of new therapeutic approaches, such as stem cell therapy, codon read-through approaches, and exon-skipping therapy.

## Supplementary Materials

Supplementary File 1
