# Supplementary material for: Concurrent Label-Free Mass Spectrometric Analysis of Dystrophin Isoform Dp427 and the Myofibrosis Marker Collagen in Crude Extracts from mdx-4cv Skeletal Muscles
Source: Proteomes. 2015 Sep 16;3(3):298–327. doi: 10.3390/proteomes3030298 (PMC5217383; doi:10.3390/proteomes3030298)
Supplement: Supplementary File 1 [file proteomes-03-00298-s001.pdf]

Supplementary Materials

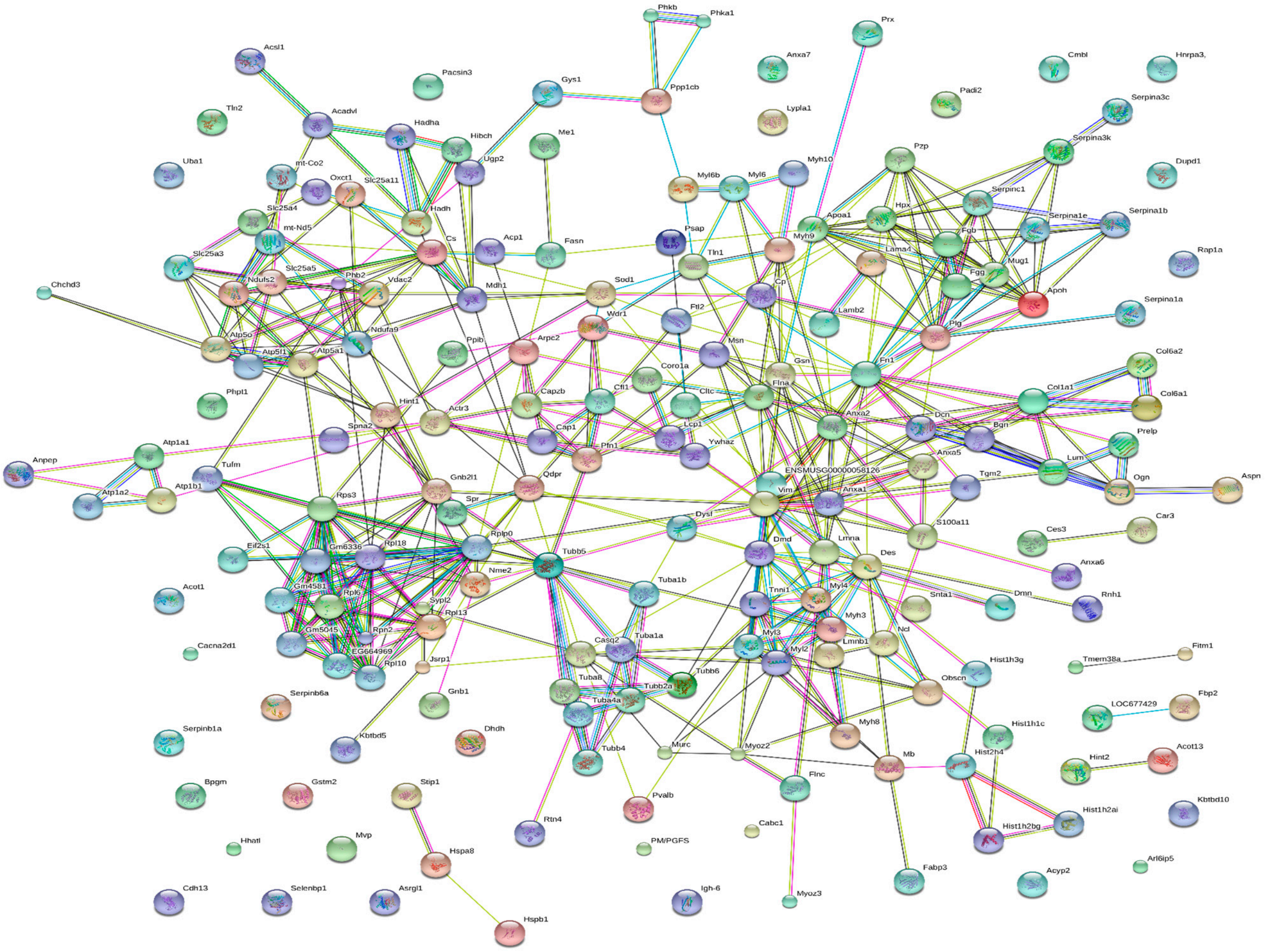

Figure S1. Bioinformatics evaluation of dystrophin isoform Dp427 and its central position in the molecular pathogenesis of X-linked muscular dystrophy. In order to generate a protein interaction map with known and predicted protein associations that include direct physical and indirect functional protein linkages, the bioinformatics STRING database [1,2] was used to analyse mass spectrometrically-identified proteins with a changed abundance in *mdx-4cv* skeletal muscles (Tables 1 and 2).

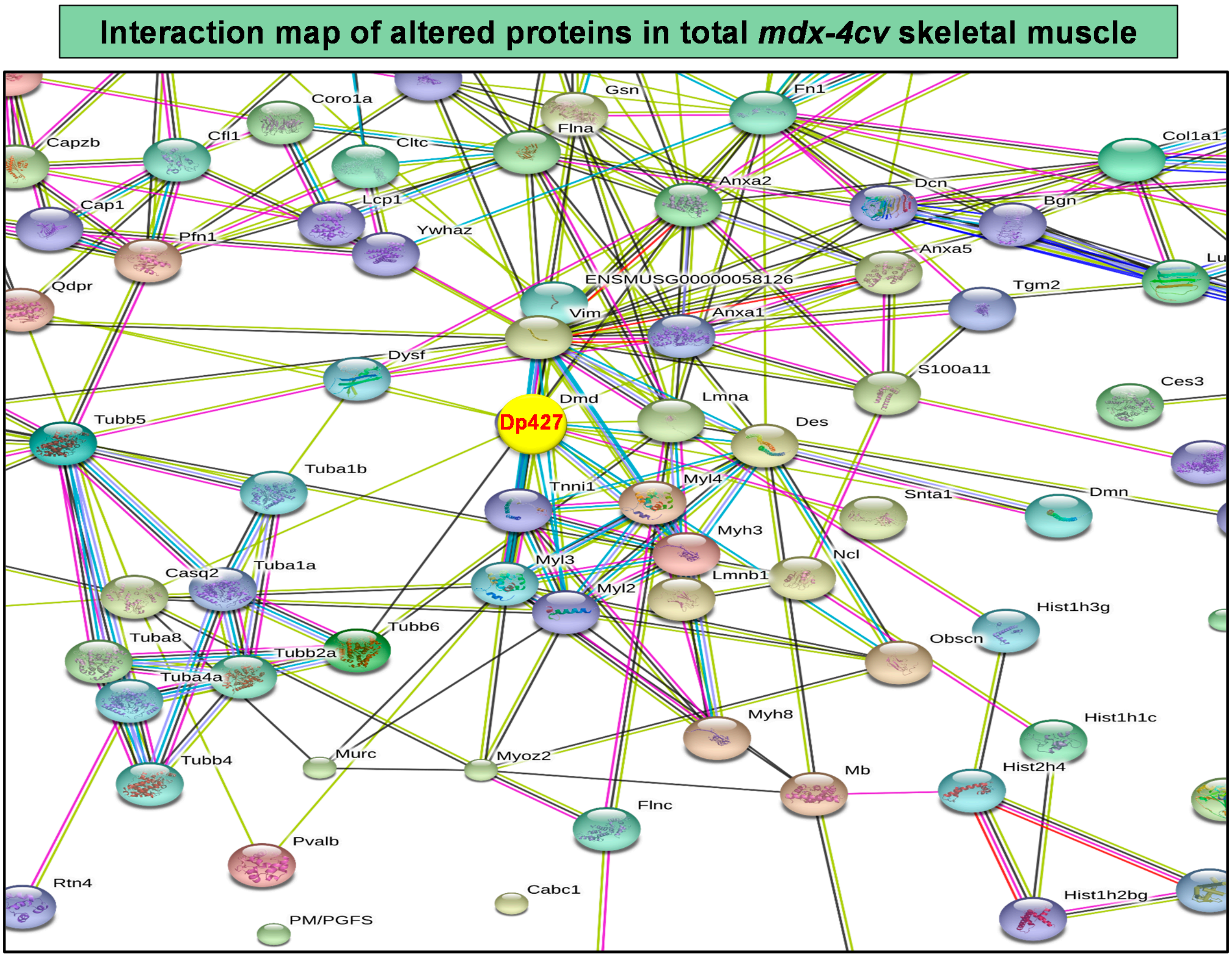

Figure S2. Focus of the bioinformatics evaluation of dystrophin isoform Dp427 and its central position in the molecular pathogenesis of X-linked muscular dystrophy. Shown is the middle part of the interaction map of altered muscle-associated proteins from *mdx-4cv* hind limb muscles, focusing on the central position of the dystrophin protein Dp427 (marked in yellow). In order to generate a protein interaction map with known and predicted protein associations that include direct physical and indirect functional protein linkages, the bioinformatics STRING database [1,2] was used to analyse mass spectrometrically-identified proteins with a changed abundance in *mdx-4cv* skeletal muscles (Tables 1 and 2).

Table S1. List of identified proteins that exhibit a significantly altered concentration in crude *mdx-4cv* hind limb muscle preparations as revealed by label-free LC-MS/MS analysis. This table contains the statistical q-values of the proteins listed in Tables 1 and 2.

Accession Number	Description	Peptide Count	*q* Value	Max Fold Change	Highest Mean Condition
P09541	Myosin light chain 4	7	0.0001	12.04	*mdx-4cv*
P13020	Gelsolin	10	0.0005	4.18	*mdx-4cv*
Q00898	Alpha-1-antitrypsin 1-5	5	0.0007	187.00	*mdx-4cv*
Q61879	Myosin-10	2	0.0007	Infinity	*mdx-4cv*
Q99JB8	Protein kinase C and casein kinase II substrate protein 3	8	0.0015	2.10	*mdx-4cv*
P11531	Dystrophin Dp427	9	0.0038	14.61	*wt*
Q99JY9	Actin-related protein 3	3	0.0049	5.59	*mdx-4cv*
Q60854	Serpin B6	13	0.0050	4.00	*mdx-4cv*
Q61233	Plastin-2	6	0.0050	5.39	*mdx-4cv*
Q9D154	Leukocyte elastase inhibitor A	9	0.0052	12.59	*mdx-4cv*
A2AMM0	Muscle-related coiled-coil protein	4	0.0062	4.60	*mdx-4cv*
Q70IV5	Synemin	2	0.0062	2.44	*mdx-4cv*
Q8K0E8	Fibrinogen beta chain	11	0.0062	10.97	*mdx-4cv*
Q8R5J9	PRA1 family protein 3	2	0.0062	16.11	*mdx-4cv*
Q9DAK9	14 kDa phosphohistidine phosphatase	2	0.0068	3.02	*wt*
Q9CVB6	Actin-related protein 2/3 complex subunit 2	3	0.0068	2.92	*mdx-4cv*
Q8BVI4	Dihydropteridine reductase	5	0.0068	2.91	*wt*
Q8VCM7	Fibrinogen gamma chain	3	0.0069	11.00	*mdx-4cv*
P48678	Prelamin-A/C	25	0.0069	2.56	*mdx-4cv*
Q00623	Apolipoprotein A-I	14	0.0070	4.63	*mdx-4cv*
O09161	Calsequestrin-2	3	0.0070	3.87	*mdx-4cv*
Q68FD5	Clathrin heavy chain 1	10	0.0070	4.82	*mdx-4cv*
P97384	Annexin A11	5	0.0070	2.32	*mdx-4cv*
P14152	Malate dehydrogenase, cytoplasmic	8	0.0070	2.33	*wt*
Q922F4	Tubulin beta-6 chain	3	0.0079	59.01	*mdx-4cv*
Q99MQ4	Asporin	5	0.0079	5.43	*mdx-4cv*
P56375	Acylphosphatase-2	3	0.0079	2.40	*wt*
P26041	Moesin	5	0.0079	3.23	*mdx-4cv*
P24369	Peptidyl-prolyl cis-trans isomerase B	2	0.0081	2.73	*mdx-4cv*
P04247	Myoglobin	16	0.0084	2.93	*wt*
P68040	Guanine nucleotide-binding protein subunit beta-2-like 1	6	0.0088	2.32	*mdx-4cv*
P17563	Selenium-binding protein 1	7	0.0089	2.36	*wt*
P10107	Annexin A1	5	0.0089	4.99	*mdx-4cv*
Q8C0M9	Isoaspartyl peptidase/L-asparaginase	3	0.0093	3.98	*wt*
Q61234	Alpha-1-syntrophin	3	0.0093	3.69	*wt*
P07356	Annexin A2	9	0.0096	3.55	*mdx-4cv*
Q02788	Collagen alpha-2(VI) chain	2	0.0098	44.37	*mdx-4cv*

Table S1. *Cont.*

Accession Number	Description	Peptide Count	*q* Value	Max Fold Change	Highest Mean Condition
P99024	Tubulin beta-5 chain	4	0.0098	9.81	*mdx-4cv*
Q9D783	Kelch-like protein 40	6	0.0098	3.10	*mdx-4cv*
Q9DB60	Prostamide/prostaglandin F synthase	2	0.0098	2.06	*mdx-4cv*
Q03265	ATP synthase subunit alpha, mitochondrial	11	0.0098	2.10	*mdx-4cv*
P26039	Talin-1	3	0.0098	3.37	*mdx-4cv*
Q8R4E4	Myozenin-3	2	0.0098	2.45	*mdx-4cv*
P62874	Guanine nucleotide-binding protein G(I)/G(S)/G(T) subunit beta-1	3	0.0107	2.68	*mdx-4cv*
Q7TMM9	Tubulin beta-2A chain	12	0.0108	2.64	*mdx-4cv*
Q91WD5	NADH dehydrogenase [ubiquinone] iron-sulfur protein 2, mitochondrial	8	0.0108	2.32	*mdx-4cv*
P31001	Desmin	18	0.0108	2.09	*mdx-4cv*
P40124	Adenylyl cyclase-associated protein 1	2	0.0108	2.08	*mdx-4cv*
P62141	Serine/threonine-protein phosphatase PP1-beta catalytic subunit	2	0.0108	2.45	*mdx-4cv*
P16015	Carbonic anhydrase CA3	21	0.0108	3.44	*wt*
P61027	Ras-related protein Rab-10	3	0.0108	3.12	*mdx-4cv*
Q07076	Annexin A7	2	0.0108	2.47	*mdx-4cv*
Q60864	Stress-induced-phosphoprotein 1	3	0.0117	2.01	*wt*
P62889	60S ribosomal protein L30	4	0.0117	2.26	*mdx-4cv*
P14733	Lamin-B1	5	0.0117	2.03	*mdx-4cv*
P22752	Histone H2A type 1	4	0.0117	3.67	*mdx-4cv*
Q8VCT4	Carboxylesterase 1D	4	0.0117	5.85	*wt*
P14602	Heat shock protein beta-1	6	0.0120	2.05	*mdx-4cv*
Q64105	Sepiapterin reductase	7	0.0120	3.14	*wt*
P20152	Vimentin	15	0.0124	2.42	*mdx-4cv*
P97927	Laminin subunit alpha-4	3	0.0124	3.03	*mdx-4cv*
O55137	Acyl-coenzyme A thioesterase 1	2	0.0124	4.35	*wt*
Q8QZS1	3-hydroxyisobutyryl-CoA hydrolase, mitochondrial	3	0.0129	3.15	*wt*
P28665	Murinoglobulin-1	15	0.0129	6.51	*mdx-4cv*
Q9D358	Low molecular weight phosphotyrosine protein phosphatase	2	0.0129	2.89	*wt*
P29621	Serine protease inhibitor A3C	2	0.0131	3.66	*mdx-4cv*
P97449	Aminopeptidase N	2	0.0131	7.80	*mdx-4cv*
P11276	Fibronectin	2	0.0132	271.96	*mdx-4cv*
Q91ZJ5	UTP--glucose-1-phosphate uridylyltransferase	15	0.0132	2.08	*wt*
P50544	Very long-chain specific acyl-CoA dehydrogenase, mitochondrial	3	0.0132	2.25	*mdx-4cv*
Q9EQK5	Major vault protein	2	0.0132	3.59	*mdx-4cv*
P07759	Serine protease inhibitor A3K	9	0.0132	2.65	*mdx-4cv*

Table S1. *Cont.*

Accession Number	Description	Peptide Count	*q* Value	Max Fold Change	Highest Mean Condition
P14824	Annexin A6	5	0.0135	2.15	*mdx-4cv*
P48036	Annexin A5	8	0.0135	2.71	*mdx-4cv*
P63017	Heat shock cognate 71 kDa protein	4	0.0135	2.03	*wt*
Q61207	Sulfated glycoprotein 1	3	0.0135	3.14	*mdx-4cv*
P70695	Fructose-1,6-bisphosphatase isozyme 2	9	0.0135	3.09	*wt*
Q08642	Protein-arginine deiminase type-2	10	0.0136	3.55	*wt*
P50543	Protein S100-A11	2	0.0136	2.30	*mdx-4cv*
P47757	F-actin-capping protein subunit beta	2	0.0138	2.34	*mdx-4cv*
Q9CQQ7	ATP synthase F(0) complex subunit B1, mitochondrial	5	0.0142	2.55	*mdx-4cv*
Q9D6F9	Tubulin beta-4A chain	7	0.0143	2.79	*mdx-4cv*
Q8BZA9	Fructose-2,6-bisphosphatase TIGAR	2	0.0147	2.08	*wt*
P22599	Alpha-1-antitrypsin 1-2	3	0.0147	3.47	*mdx-4cv*
O35129	Prohibitin-2	3	0.0147	2.07	*mdx-4cv*
Q3TMP8	Trimeric intracellular cation channel type A	2	0.0147	8.94	*mdx-4cv*
P51881	ADP/ATP translocase 2	2	0.0147	8.06	*mdx-4cv*
Q9ESD7	Dysferlin	2	0.0148	16.08	*mdx-4cv*
P28653	Biglycan	4	0.0148	17.82	*mdx-4cv*
A2AAJ9	Obscurin	9	0.0156	2.27	*mdx-4cv*
P18760	Cofilin-1	5	0.0156	2.90	*mdx-4cv*
O89053	Coronin-1A	2	0.0156	8.10	*mdx-4cv*
P08228	Superoxide dismutase [Cu-Zn]	5	0.0156	2.09	*wt*
P01872	Ig mu chain C region	3	0.0156	3.65	*mdx-4cv*
Q61838	Alpha-2-macroglobulin	28	0.0156	3.79	*mdx-4cv*
O88342	WD repeat-containing protein 1	3	0.0156	2.05	*mdx-4cv*
P32261	Antithrombin-III	3	0.0157	2.66	*mdx-4cv*
P70349	Histidine triad nucleotide-binding protein 1	6	0.0157	2.27	*wt*
Q8BK84	Dual specificity phosphatase DUPD1	2	0.0157	2.21	*mdx-4cv*
Q8BFR5	Elongation factor Tu, mitochondrial	9	0.0158	2.37	*mdx-4cv*
P62835	Ras-related protein Rap-1A	2	0.0158	10.62	*mdx-4cv*
P14869	60S acidic ribosomal protein P0	3	0.0158	2.57	*mdx-4cv*
P09405	Nucleolin	2	0.0158	2.75	*mdx-4cv*
Q9JK53	Prolargin	5	0.0159	2.57	*mdx-4cv*
P29391	Ferritin light chain 1	9	0.0164	2.68	*mdx-4cv*
P06801	NADP-dependent malic enzyme	8	0.0166	2.85	*wt*
Q8R1G2	Carboxymethylenebutenolidase homolog	4	0.0168	2.64	*wt*
Q61147	Ceruloplasmin	2	0.0170	4.09	*mdx-4cv*
Q9WTR5	Cadherin-13	3	0.0171	2.37	*mdx-4cv*
P19096	Fatty acid synthase	3	0.0174	4.00	*wt*
P68368	Tubulin alpha-4A chain	3	0.0176	2.28	*mdx-4cv*
P68369	Tubulin alpha-1A chain	12	0.0177	3.68	*mdx-4cv*
Q6ZWY9	Histone H2B type 1-C/E/G	3	0.0178	3.46	*mdx-4cv*

Table S1. *Cont.*

Accession Number	Description	Peptide Count	*q* Value	Max Fold Change	Highest Mean Condition
Q8BTM8	Filamin-A	3	0.0180	2.98	*mdx-4cv*
P16546	Spectrin alpha chain, non-erythrocytic 1	5	0.0186	3.75	*mdx-4cv*
P63101	14-3-3 protein zeta/delta	4	0.0186	2.23	*mdx-4cv*
Q8CI43	Myosin light chain 6B	2	0.0196	2.89	*mdx-4cv*
Q99P72	Reticulon-4	2	0.0196	3.96	*mdx-4cv*
Q9D0S9	Histidine triad nucleotide-binding protein 2, mitochondrial	2	0.0199	2.15	*wt*
P18826	Phosphorylase b kinase regulatory subunit alpha, skeletal muscle isoform	3	0.0200	3.35	*mdx-4cv*
A2AUC9	Kelch-like protein 41	12	0.0201	2.97	*mdx-4cv*
Q9CRB9	Coiled-coil-helix-coiled-coil-helix domain-containing protein 3, mitochondrial	2	0.0202	2.28	*wt*
Q8VDD5	Myosin-9	4	0.0213	15.76	*mdx-4cv*
E9PZQ0	Ryanodine receptor 1	27	0.0213	3.72	*mdx-4cv*
Q9CQR4	Acyl-coenzyme A thioesterase 13	2	0.0220	2.14	*wt*
Q9D1G3	Protein-cysteine N-palmitoyltransferase HHAT-like protein	5	0.0221	6.55	*mdx-4cv*
O89104	Synaptophysin-like protein 2	2	0.0222	3.73	*mdx-4cv*
Q9D0K2	Succinyl-CoA:3-ketoacid coenzyme A transferase 1, mitochondrial	2	0.0222	2.61	*wt*
Q9DB20	ATP synthase subunit O, mitochondrial	4	0.0226	2.19	*mdx-4cv*
Q60605	Myosin light polypeptide 6	5	0.0227	2.10	*mdx-4cv*
P62806	Histone H4	5	0.0227	3.28	*mdx-4cv*
P14094	Sodium/potassium-transporting ATPase subunit beta-1	2	0.0227	4.39	*mdx-4cv*
Q6PIE5	Sodium/potassium-transporting ATPase subunit alpha-2	6	0.0229	5.02	*mdx-4cv*
P15864	Histone H1.2	2	0.0230	3.28	*mdx-4cv*
P47911	60S ribosomal protein L6	2	0.0232	2.59	*mdx-4cv*
P11404	Fatty acid-binding protein FABP3, heart	5	0.0232	2.21	*wt*
Q8BMS1	Trifunctional enzyme subunit alpha, mitochondrial	9	0.0232	3.25	*mdx-4cv*
O08532	Voltage-dependent calcium channel subunit alpha-2/delta-1	5	0.0235	3.03	*mdx-4cv*
P68433	Histone H3.1	3	0.0235	5.98	*mdx-4cv*
Q91V79	Fat storage-inducing transmembrane protein 1	2	0.0241	5.15	*mdx-4cv*
Q61425	Hydroxyacyl-coenzyme A dehydrogenase, mitochondrial	2	0.0244	2.30	*mdx-4cv*
P14148	60S ribosomal protein L7	3	0.0250	3.23	*mdx-4cv*
P15327	Bisphosphoglycerate mutase	3	0.0250	2.02	*wt*
Q61292	Laminin subunit beta-2	5	0.0250	2.08	*mdx-4cv*
Q91VI7	Ribonuclease inhibitor	7	0.0250	2.49	*mdx-4cv*
P13542	Myosin-8	4	0.0255	8.63	*mdx-4cv*

Table S1. *Cont.*

Accession Number	Description	Peptide Count	*q* Value	Max Fold Change	Highest Mean Condition
Q8BH59	Calcium-binding mitochondrial carrier protein Aralar1	7	0.0256	3.91	*mdx-4cv*
P07758	Alpha-1-antitrypsin 1-1	9	0.0256	2.94	*mdx-4cv*
Q9DC69	NADH dehydrogenase [ubiquinone] 1 alpha subcomplex subunit 9, mitochondrial	3	0.0257	2.26	*mdx-4cv*
O55103	Periaxin	2	0.0259	2.67	*wt*
Q9CZU6	Citrate synthase, mitochondrial	5	0.0261	2.07	*mdx-4cv*
Q8VEM8	Phosphate carrier protein, mitochondrial	5	0.0261	3.22	*mdx-4cv*
Q8VDN2	Sodium/potassium-transporting ATPase subunit alpha-1	5	0.0262	6.07	*mdx-4cv*
P00405	Cytochrome c oxidase subunit 2	3	0.0265	3.52	*mdx-4cv*
P62962	Profilin-1	4	0.0265	2.49	*mdx-4cv*
P62908	40S ribosomal protein S3	3	0.0265	4.16	*mdx-4cv*
P09542	Myosin light chain 3	8	0.0267	3.51	*wt*
P05213	Tubulin alpha-1B chain	2	0.0267	2.30	*mdx-4cv*
Q62000	Mimecan	7	0.0267	2.42	*mdx-4cv*
Q01339	Beta-2-glycoprotein 1	3	0.0269	4.11	*mdx-4cv*
P11087	Collagen alpha-1(I) chain	4	0.0271	5.92	*mdx-4cv*
P28654	Decorin	5	0.0271	3.10	*mdx-4cv*
Q9JJW5	Myozenin-2	2	0.0271	3.98	*wt*
Q91X72	Hemopexin	13	0.0273	2.58	*mdx-4cv*
P48962	ADP/ATP translocase 1	9	0.0274	2.86	*mdx-4cv*
P97823	Acyl-protein thioesterase 1	2	0.0274	3.96	*wt*
Q9DBG6	Dolichyl-diphosphooligosaccharide–protein glycosyltransferase subunit 2	2	0.0277	4.26	*mdx-4cv*
Q04857	Collagen alpha-1(VI) chain	3	0.0278	3.12	*mdx-4cv*
P13541	Myosin-3	6	0.0278	2.71	*mdx-4cv*
P35980	60S ribosomal protein L18	3	0.0279	2.42	*mdx-4cv*
P20918	Plasminogen	2	0.0281	3.89	*mdx-4cv*
Q6ZWX6	Eukaryotic translation initiation factor 2 subunit 1	2	0.0282	2.28	*mdx-4cv*
P03921	NADH-ubiquinone oxidoreductase chain 5	3	0.0285	6.28	*mdx-4cv*
Q9Z1E4	Glycogen [starch] synthase, muscle	4	0.0291	2.33	*mdx-4cv*
P15626	Glutathione S-transferase Mu 2	5	0.0291	2.13	*wt*
Q8R429	Sarcoplasmic/endoplasmic reticulum calcium ATPase 1	39	0.0294	2.78	*mdx-4cv*
P41216	Long-chain-fatty-acid–CoA ligase 1	5	0.0296	5.19	*mdx-4cv*
P35979	60S ribosomal protein L12	3	0.0296	2.02	*mdx-4cv*
Q3MI48	Junctional sarcoplasmic reticulum protein 1	3	0.0305	2.18	*mdx-4cv*
P51885	Lumican	5	0.0305	2.07	*mdx-4cv*
P47963	60S ribosomal protein L13	2	0.0325	2.31	*mdx-4cv*
P21981	Protein-glutamine gamma-glutamyltransferase 2	2	0.0325	8.77	*mdx-4cv*
Q9JJZ2	Tubulin alpha-8 chain	2	0.0328	2.10	*mdx-4cv*

Table S1. *Cont.*

Accession Number	Description	Peptide Count	*q* Value	Max Fold Change	Highest Mean Condition
P21107	Tropomyosin alpha-3 chain	3	0.0333	2.72	*wt*
Q71LX4	Talin-2	2	0.0333	3.69	*mdx-4cv*
Q9CZM2	60S ribosomal protein L15	2	0.0335	2.52	*mdx-4cv*
Q9DBB8	Trans-1,2-dihydrobenzene-1,2-diol dehydrogenase	2	0.0348	2.90	*wt*
Q60936	Chaperone activity of bc1 complex-like, mitochondrial	2	0.0367	2.02	*mdx-4cv*
Q01768	Nucleoside diphosphate kinase B	3	0.0377	2.21	*wt*
P51667	Myosin regulatory light chain 2, ventricular/cardiac muscle isoform	6	0.0377	3.64	*wt*
Q02053	Ubiquitin-like modifier-activating enzyme 1	2	0.0388	2.01	*mdx-4cv*
Q9CR62	Mitochondrial 2-oxoglutarate/malate carrier protein	2	0.0392	4.60	*mdx-4cv*
Q9WUZ5	Troponin I, slow skeletal muscle	2	0.0403	2.50	*wt*
Q6ZWV3	60S ribosomal protein L10	2	0.0424	4.12	*mdx-4cv*
Q7TSH2	Phosphorylase b kinase regulatory subunit beta	2	0.0425	5.50	*mdx-4cv*
Q8VHX6	Filamin-C	20	0.0444	2.07	*mdx-4cv*
P32848	Parvalbumin alpha	5	0.0457	3.95	*wt*
Q8BG05	Heterogeneous nuclear ribonucleoprotein A3	2	0.0458	2.28	*mdx-4cv*
Q60930	Voltage-dependent anion-selective channel protein 2	9	0.0460	2.37	*mdx-4cv*

Reference

1. STRING Functional Protein Association Network. Available online: http://string-db.org/ (accessed on 3 February 2015).
2. Franceschini, A.; Szklarczyk, D.; Frankild, S.; Kuhn, M.; Simonovic, M.; Roth, A.; Lin, J.; Minguez, P.; Bork, P.; von Mering, C.; *et al.* STRING v9.1: Protein-protein interaction networks, with increased coverage and integration. *Nucleic Acids Res.* **2013**, *41*, D808–D815.
